# The association of Dietary Approach to Stop Hypertension (DASH) diet with metabolic healthy and metabolic unhealthy obesity phenotypes

**DOI:** 10.1038/s41598-019-55285-6

**Published:** 2019-12-10

**Authors:** Hossein Farhadnejad, Mina Darand, Farshad Teymoori, Golaleh Asghari, Parvin Mirmiran, Fereidoun Azizi

**Affiliations:** 1 Nutrition and Endocrine Research Center, Research Institute for Endocrine Sciences, Shahid Beheshti University of Medical Sciences, Tehran, I.R. Iran; 2 Department of Nutrition, School of Public Health, Iran University of Medical Sciences, Tehran, Iran; 3 Endocrine Research Center, Research Institute for Endocrine Sciences, Shahid Beheshti University of Medical Sciences, Tehran, I.R. Iran

**Keywords:** Obesity, Nutrition

## Abstract

The current study aimed at investigating the association between Dietary Approach to Stop Hypertension (DASH) diet and odds of obesity phenotypes, is a cross sectional study conducted on 3218 Iranian overweight or obese participants (BMI >25 kg/m^2^), aged ≥20 years, who participated in the fourth phase (2009–2011) of the Tehran Lipid and Glucose Study. Using a valid and reliable food-frequency questionnaire, DASH diet scores between 8 and 40 points were determined. Obesity phenotypes including metabolic unhealthy obesity (MUHO) and metabolic healthy obesity (MHO) were defined using criteria of the Joint International statement(JIS) for metabolic syndrome. Multivariable logistic regression was used to determine the odds ratio (OR) for obesity phenotypes according to the tertiles of the DASH diet. Mean ± SD age of participants (43.5% male) was 39.2 ± 9.5 years and median (25–75 interquartile range) DASH diet score was 24 (21–27); percentages of MHO and MUHO subjects were 33.4 and 66.6%, respectively. In the multivariable adjusted model, after controlling for age, sex, BMI, physical activity, smoking status, socioeconomic status, and energy intake, participants in the highest tertile of DASH diet had lower odds of MUHO (OR:0.79;95%CI:0.64–0.98), in comparison to those in the lowest one (P for trend = 0.040). Our findings indicate that adherence to DASH diet may be favourable in prevention of metabolic abnormalities in overweight and obese individuals.

## Introduction

Obesity represents a rapidly growing public health problem worldwide over the last few decades^1^. Epidemiological studies have shown that combinations of obesity and metabolic abnormalities lead to increased risk of chronic diseses, such as diabetes, cardiovascular diseases (CVD), and all-cause mortality^2,3^. Recently, it has been shown that even individuals in the same body mass index category can have different levels of metabolic components, including lipid profiles, blood glucose, blood pressure, and waist circumference^3^. Among obese individuals, two phenotypes, including subjects with normal metabolic characteristics as the metabolically healthy obese (MHO) phenotype, and those with metabolically unhealthy status as the metabolically unhealthy obese (MUHO) phenotypes have been defined^4,5^, the long-term health outcomes of the MHO and MUHO phenotypes are still not fully understood^6^; one study reported that both metabolically healthy and unhealthy obese individuals are faced with increased risk of CVD and mortality^7^; however, the Hamer *et al*. study indicates that compared with the metabolically healthy nonobese participants, MHO was not associated with increased risk of CVD and all cause mortality^8^. Also, some evidence shows that the metabolically unhealthy obese phenotype had higher risk of chronic diseases, compared to the healthy one^9^.

Dietary patterns along with physical activity are important environmental factors that contribute to the progression of obesity and metabolic disorders^10,11^. Considering that meals, include a wide variety of foods with various mixtures of nutrients, which may have interactive effects, investigating dietary patterns is essential for providing valuable information, beyond individual nutrients or food groups^12^. The Dietary Approach to Stop Hypertension (DASH) diet, a dietary pattern, currently receiving much attention, was originally designed to reduce blood pressure^13^; this diet emphasizes intakes of fruits, vegetables, whole grains, nuts, legumes, moderate amounts of low-fat dairy; and underscores reduced intakes of red or processed meats, sodium, and sweetened beverages^13^.

Some studies with controversial results have explored the association between healthy diet indices such as DASH diet, Mediterranean diet, and food pyramid compliance with odds of obesity phenotypes^14,15^; although greater adherence to the Mediterranean was positively associated with MHO, compared to MUHO, and higher adherence to DASH diet score was related with lower odds of metabolically unhealthy normal weight phenotype in comparison to metabolically healthy normal weight younger adults, no significant association of Mediterranean diet and DASH index with obesity phenotypes was found in the elderly^15^. It has been reported that higher compliance to food pyramid recommendations but not DASH score is favorably related to MHO^14^. Since prevention of obesity-related cardiometabolic diseases is an enormous medical and socioeconomic task, and extremely important in terms of cost effectiveness^16^, the determination and assessment of enviromental factors such as dietary patterns which protect obese individuals against obesity-related metabolic diseases is vital. We therefore aim to investigate associations of higher adherence to the DASH diet with metabolically healthy and unhealthy obesity phenotypes among an Iranian population.

## Materials and Methods

### Subjects

The current study was conducted within the framework of the Tehran Lipid and Glucose Study (TLGS), a population-based prospective study, aimed at determining the risk factors for non-communicable diseases among a representative urban population of Tehran, including 15005 participants, aged ≥3 years. The first survey of TLGS began in 1999 and data collection, conducted at 3-year intervals, is ongoing. The baseline survey was a cross-sectional study, and surveys II, III, IV, and V were prospective follow-up surveys^17,18^.

**Figure 1:**
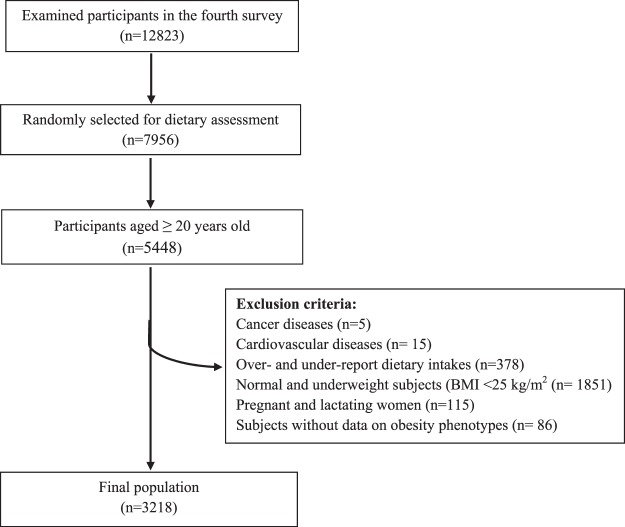
Flow chart of the Tehran Lipid and Glucose Study (TLGS) participants.

In the fourth survey of the TLGS (2009–2011), from among 12823 participants, 7956 randomly selected subjects, agreed to complete dietary assessment. For the present study, of 5448 individuals, aged ≥20 years, after exclusion of normal and underweight subjects (BMI <25 kg/m^2^; n = 1851), those with prevalent cancer (n = 5), cardiovascular diseases (n = 15), pregnant and lactating women (n = 115), participants with under- or over-reported dietary intakes below 800 kcal/d or above 4200 kcal/d, respectively (n = 378), and those without data on obesity phenotypes (n = 86), 3218 participants were finally enrolled in the study, some of whom fell into more than one category (Fig. 1).

Written informed consents were obtained from all participants and the study protocol was reviewed and approved by the ethics research council of the Research Institute for Endocrine Sciences, Shahid Beheshti University of Medical Sciences. The study was performed in accordance with the Declaration of Helsinki as well as our institutional guidelines.

### Measurements

Details of the study method and measurements have been reported previously^18–21^. Dietary data over the previous year were collected using a valid and reliable semi-quantitative food frequency questionnaire at baseline^22^. Trained dieticians asked participants to designate their consumption frequency for each food item during the previous year on a daily, weekly or monthly basis; portion sizes of consumed foods, reported in household measures, were then converted to grams^21^. DASH diet score in the Fung *et al*. study^23^ was determined based on eight components, i.e. higher intakes of whole grains, fruits, vegetables, legumes and nuts, and low-fat dairy and lower intakes of red or processed meats, sodium, and sweetened beverages. The intake of each food component was energy adjusted (g/1000 kcal) and then categorized into quintiles. Subject’s quintile rankings were used for whole grains, fruits, vegetables, legumes and nuts, and low-fat dairy to determine component scores, e.g. participants in the lowest quintile were recieved 1 point, and those in the highest one were recieved 5 points. For sodium, red and processed meats and sugar-sweetened beverages, those in the lowest quintile were assigned 5 points, and those in the highest one were assigned 1 point. We then summed up the scores for all the eight components to compute the DASH score. Thus, the total DASH score ranged from 8 (minimum adherence) to 40 (maximal adherence).

A trained interviewer used a pretested questionnaire to collect data on age, sex, medical history, medication use, and smoking habits. Smoking status was defined as nonsmoker and smoker (ex-smoker, current or occasionally). Education level was categorized based on years of education (≤12 years, >12 years). To collect physical activity, we used the Modifiable Activity Questionnaire (MAQ), which has been previously modified and validated among Iranians^24^. The participant’s weight, height, waist circumference (WC), systolic blood pressure (SBP), and diastolic blood pressure (DBP) were measured based on standard protocols as previously described^19,21^. Body mass index was computed as weight (kg) divided by height (m^2^). A blood sample was taken at the TLGS research laboratory in a sitting position after 12–14 h of overnight fasting according to the standard protocol to measure fasting plasma glucose (FPG), triglyceride (TG), total cholesterol (TC), high density lipoprotein cholesterol (HDL-C), and low density lipoprotein cholesterol (LDL-C)^19,20^.

### Definitions

Obesity phenotypes, including MUHO and MHO were defined based on the Joint Interim Statement (JIS) criteria for metabolic syndrome (MetS)^25^. Participants were considered as having MUHO if they had ≥2 of the following: WC ≥ 91 cm in women and ≥89 cm in men^26^; FPG ≥ 100 mg/dl or drug treatment; fasting TGs ≥ 150 mg/dl or drug treatment; fasting HDL-C <50 mg/dl in women and <40 mg/dl in men or drug treatment; raised blood pressure defined as SBP ≥ 130 mmHg/DBP ≥ 85 mmHg or antihypertensive drug treatment. Those who had ≤1 component were categorized as MHO.

### Statistical analyses

Data analysis was performed using the SPSS software (Statistical Package for the Social Sciences, version 15.0, SPSS Inc, Chicago, IL, USA). Distribution of variables was checked using histogram chart and the Kolmogorov-Smirnov’s test. Participants were categorized according to tertiles of DASH diet score cutoff points (≤22, 23–26, and ≥27). Baseline characteristics of participants are expressed as mean ± SD or median (25–75 interquartile range) for continuous variables, and percentages for categorical variables across tertiles of DASH score. We have used linear regression and Chi square to test the trend of continuous and dichotomous variables across tertiles of DASH score, respectively, and P for trend was reported. Odds of MUHO in comparison to reference category (MHO) across tertiles of DASH score was assessed using logistic regression models, adjusted for potential confounders including age, sex, BMI, physical activity, smoking status, dietary energy intake, and education level. We also analyzed the association of DASH score as a continuous variable with odds of MUHO in our population. Odds ratio (OR) and 95% confidence interval (CI) were reported, and P-values < 0.05 were considered statistically significant.

## Results

The mean ± SD age of participants (43.5% male) was 39.2 ± 9.5 years and median (25–75 interquartile range) of the DASH diet score was 24 (21–27). Percentages of MHO and MUHO were 33.4 and 66.6%, respectively.

General characteristics of the study population across tertiles of DASH diet score are presented in Table 1. Participants in the highest DASH diet tertile were more likely to be female, older, have higher physical activaty and HDL-C concentration, and less likely to be smokers, compared to those in the lowest one (P < 0.05). There were no significant differences in any other demographic, anthropometric, and biochemical measures across tertiles of DASH diet scores.

**Table 1:** Characteristics of participants across tertiles of DASH diet score in 3218 participants of the Tehran Lipid and Glucose Study.

Characteristics	Tertiles of DASH score			*P* value^*^
	Tertile 1 (n = 1220)	Tertile 2 (n = 1070)	Tertile 3 (n = 928)	
Age (years)	37.3 ± 9.0	39.6 ± 8.8	41.2 ± 8.9	<0.001
Men (%)	50.7	44.1	33.4	<0.001
Body mass index (kg/m^2^)	29.5 ± 3.7	29.5 ± 3.9	29.7 ± 3.7	0.139
Smoking (%)	15.4	10.5	8.8	<0.001
Physical activity (Met-h/day)	74.8 (41.6–107.0)	70.7 (38.7–99.8)	62.7 (33.7–98.1)	<0.001
Waist circumference (cm)	97.4 ± 10.1	96.8 ± 9.9	96.7 ± 9.7	0.074
Fasting blood glucose (mg/dl)	96.2 ± 19.0	98.0 ± 22.5	98.0 ± 26.1	0.072
Triglycerides (mg/dl)	151.8 ± 87.9	161.0 ± 164.4	148.8 ± 87.9	0.573
HDL- cholesterol (mg/dl)	44.9 ± 10.6	45.8 ± 10.9	47.1 ± 11.4	<0.001
Systolic blood pressure (mmHg)	113.7 ± 14.0	114.4 ± 14.1	113.4 ± 14.3	0.674
Diastolic blood pressure (mmHg)	77.4 ± 10.1	78.2 ± 10.9	77.1 ± 10.8	0.529
Education level (>12 years) (%)	27.7	29.3	28.7	0.351

Data are presented as mean ± standard deviation or median (interquartile range) and as percentage.

*Significant differences (p < 0.05) was obtained using chi square for categorical variables and linear regression for continuous variables.

Dietary intakes of participants according to DASH diet score are shown in Table 2; participants in the highest tertile of DASH diet score had higher intakes of vegetables, whole grain, fruits, nuts and legumes, and low-fat dairy, but lower intakes of sweetened beverages and red and processed meat. Dietary intakes of total energy, carbohydrates, proteins, fiber, folate, potassium, and calcium were significantly increased across DASH diet tertiles (P < 0.001), whereas intakes of total fat, saturated fat, monounsaturated fat, polyunsaturated fat, and sodium were decreased (P < 0.001).

**Table 2:** Dietary nutrients and food group intake across tertiles of DASH diet score in 3218 participants of the Tehran Lipid and Glucose Study.

Nutrient and food groups	Tertiles of DASH score			P for trend
	Tertile 1 (n = 1220)	Tertile 2 (n = 1070)	Tertile 3 (n = 928)	
**DASH score (median)**	20.0	24.0	29.0	
Vegetables (g/1000 Kcal)	112.2 ± 60.0	147.1 ± 80.4	192.8 ± 108.9	<0.001
Fruits (g/1000 Kcal)	117.5 ± 101.1	162.6 ± 114.7	229.3 ± 151.6	<0.001
Whole grains (g/1000 Kcal)	52.3 ± 41.6	60.9 ± 41.4	62.7 ± 38.8	<0.001
Nuts and legumes (g/1000 Kcal)	17.8 ± 13.2	21.7 ± 14.4	27.6 ± 18.6	<0.001
Low-fat dairy (g/1000 Kcal)	83.7 ± 68.7	112.4 ± 81.1	132.6 ± 86.1	<0.001
sweetened beverages (g/1000 Kcal)	19.3 (8.1–83.0)	9.5 (3.5–19.8)	4.1 (0.77–11.3)	<0.001
Red and processed meat (g/1000 Kcal)	12.2 ± 8.6	8.9 ± 6.3	6.8 ± 6.0	<0.001
Total energy (kcal/day)	2292 ± 697	2430 ± 728	2585 ± 701	<0.001
Carbohydrates (% of energy)	57.5 ± 6.6	58.3 ± 6.4	60.3 ± 12.8	<0.001
Proteins (% of energy)	14.4 ± 3.1	15.0 ± 3.3	15.7 ± 12.9	<0.001
Fat (% of energy)	30.8 ± 6.5	30.3 ± 5.9	29.0 ± 5.7	<0.001
Saturated fat (% of energy)	10.0 ± 2.8	9.9 ± 2.5	9.4 ± 2.7	<0.001
Monounsaturated fat (% of energy)	10.4 ± 3.0	10.1 ± 2.5	9.6 ± 3.1	<0.001
Polyunsaturated fat(% of energy)	6.2 ± 2.0	6.0 ± 1.9	5.8 ± 2.4	<0.001
Dietary fiber (g/1000Kcal)	18.1 ± 7.0	19.5 ± 6.3	21.4 ± 6.1	<0.001
Folate (µg/1000Kcal)	245 ± 43	251 ± 40	259 ± 42	<0.001
Sodium (mg/1000Kcal)	1559 ± 459	1525 ± 393	1430 ± 362	<0.001
Potassium (mg/1000 kcal)	1677 ± 415	1977 ± 488	2265 ± 532	<0.001
Calcium (mg/1000Kcal)	547 ± 167	637 ± 196	689 ± 204	<0.001

Data values are mean ± SD or median (interquartile range).

The ORs (95% CI) of MUHO across tertile categories of DASH diet score are presented in Table 3. In the age and sex-adjusted model, the odds of MUHO in the third tertile of DASH diet score was 0.85 (95% CI: 0.69–1.04; P for trend = 0.128). After additional adjustment for energy intake, physical activity, smoking, BMI, and education level, the odds of MUHO in the highest, compared to the lowest DASH diet score category, decreased by 21% (OR: 0.79, 95% CI: 0.64–0.98; P for trend = 0.040). Also, we investigated the association of DASH score as a continuous variable with odds of obesity phenotypes in our population, and the statistical significance of findings was attenuated: the odds of MUHO, in comparison to MHO, based on one score change in the DASH diet was small (OR: 0.91, 95% CI: 0.85–1.01; P for trend = 0.100), (Table 4).

**Table 3:** Multivariable-adjusted odds ratios (and 95% CIs) for obesity phenotype (MHO in comparison to MUHO) across tertiles of DASH score in 3218 participants of the Tehran Lipid and Glucose Study.

Characteristics	Tertiles of DASH score			*P* trend
	T1(lowest)	T2	T3(highest)	
MUHO/Total population	815/1220	728/1070	599/928	
Model 1^*^	1.00 (Ref)	0.86 (0.72–1.03)	0.86 (0.72–1.03)	0.119
Model 2^†^	1.00 (Ref)	0.86 (0.70–1.04)	0.85 (0.69–1.04)	0.128
Model 3^‡^	1.00 (Ref)	0.86 (0.70–1.06)	0.79 (0.64–0.98)	0.040

^*^Crude model.

^†^Adjusted for age and sex.

^‡^Additionally adjusted for dietary energy intake, physical activity, smoking, body mass index, and education level.

MHO: Metabolically healthy obese; MUHO: Metabolically unhealthy obese

**Table 4:** Multivariable-adjusted odds ratios (and 95% CIs) for obesity phenotype per each score increase in the DASH score in 3218 participants of the Tehran Lipid and Glucose Study.

Characteristics	per each score increase in the DASH score	*P* value
	OR (95% CI)	
Model 1^*^	0.97 (0.90–1.07)	0.503
Model 2^†^	0.96 (0.88–1.04)	0.347
Model 3^‡^	0.91 (0.85–1.01)	0.100

^*^Crude model.

^†^Adjusted for age and sex.

^‡^Additionally adjusted for dietary energy intake, physical activity, smoking, body mass index, and educational level.

## Discussion

In this cross- sectional study, conducted on an adult Tehranian population, we found that, compared to MHO, greater adherence to the DASH diet was associated with 21% lower odds of MUHO, independent of age, sex, energy intake, physical activity, BMI, smoking, and educational level. Considering that the current study has been conducted on a large sample of Tehranian adults population, our findings on positive associaion of DASH diet with healthy metabolic status are potentially generalizable to the Iranian adult population.

In the current study, the association of DASH score as a continuous variable has been assessed with odds of obesity phenotypes in our population. Considering that the DASH score is a discrete variable with a narrow range between 8–40 (the range of this score in our population was from 9 to 37). When we analyzed the DASH score as a continuous variable, the statistical significance of findings was attenuated and our results no longer remained significant. It should be noted that in the epidemiological studies, variation in the exposure variable among the study population is so important and for simplicity of interpretation of results and their clinical applicability, studies mainly focus on high and poor quality diet, which can be attained by categorizing the exposure variable. Therefore, similar to more than 90% of other nutritional studies, we categorized DASH score in to tertiles in addition to the using it as a continuous variable. Categorizing DASH diet is helpful to determine more considerable associations with metabolic health status in obese participants.

Previously, the DASH-style diet, Mediterranean diet, healthy eating index (HEI), and food pyramid recommendation scores have been assessed with metabolic status in obese individuals and controversial findings were reported^14,15,27^. The Camhi *et al*. study indicates that HEI significantly differed between MHO and MUHO phenotypes among women aged 19–44 years; however, no significant difference was observed among men and elderly women^27^. Phillips *et al*. revealed that greater adherence to Irish food pyramid recommendations was positively related to metabolic health in MHO participants^14^; however, no significant association of DASH score with metabolic health was observed among obese participants^15^. Park *et al*. study^15^ indicate that greater adherence to DASH style diet plays favourable role in reducing metabolic abnormalities in normal weight individuals, findings observed among younger participants, not older ones; however, we found significant association between DASH diet and metabolic status in obese participants aged ≥ 20 years.

Although, limited studies have been conducted on the association of DASH diet with obesity or metabolic abnormalities, their results are in agreement to those of ours^28,29^. In the Saneei *et al*. study, adherence to the DASH diet was inversely related to metabolic abnormalities including central obesity, hypertriglyceridemia, high blood pressure, and low HDL-C concentration^29^. Similarly, Azadbakht *et al*. showed that higher adherence to the DASH diet was inversely associated with an unhealthy metabolic status^28^. It has been reported that obese individuals can have different levels of metabolic components, such as lipid profiles, glucose tolerance, blood pressure, and waist circumference^3^, heterogeneity in which various factors such as dietary patterns can play important role. Therefore, we examined participants in terms of metabolic disorders as well as overweight and obesity, based on a standard definition in two groups, the MHO and MUHO, and findings showed the DASH diet to be inversely associated with unhealthy metabolic status in overweight and obese individuals. The DASH style diet emphasizes increased intakes of fruits, vegetables, whole grains, nuts and legumes, low-fat dairy, and dietary fiber, and reduced intakes of red meat and sugar-sweetened beverages, all of which are closely associated with decrease in risk of metabolic abnormalities^30–35^. In the Yoo *et al*. study, individuals with higher intakes of fruit and vegetables had lower prevalence of MetS^34^. Similarly, it has been shown that higher intakes of whole grain, nuts, total fiber, fruit fiber, and legume fiber decreases the risk of metabolic abnormalities^31,35^. Furthermore, some studies have shown inverse associations of dairy products and calcium intakes with MetS^30,33^. Evidence shows the association of red meat and sugar-sweetened beverages with risk of metabolic abnormalities and inflammation^36,37^.

Although it is not clear how the DASH diet may modulate the odds of MHO, potential mechanisms linking DASH diet or its components with metabolic abnormalities have been previously reported^30,38–40^. It is suggested that high dietary fibre attenuates indicators of metabolic abnormalities such as FPG, TGs, BP and WC^38^; another mechanism suggested is the higher intakes of low-fat dairy via the effects of calcium on insulin sensitivity, WC, BP, and lipid profiles^30,40^. Also, the DASH dietry pattern contains high levels of potassium, magnesium, vitamin C, and phytochemicals, which reduce the risk of insulin resistance, obesity, and MetS^39^.

The current study has several strengths. This study is the first large size population-based study to examine the association between DASH diet and different phenotypes of obesity in the Middle East and North Africa (MENA) region. In the current study, we also used valid and reliable food-frequency and physical activity questionnaires for the population. However, our study does have some limitations. The cross-sectional nature of the study limited us from establishing a cause and effect relation between DASH diet and obesity phenotypes. Also, despite adjusting for the confounding role of various variables in our analysis, residual confounding due to unknown or unmeasured confounders, such as socioeconomic status cannot be excluded.

Our findings show that DASH diet score may be associated with decreased the odds of MUHO in overweight and obese individuals, indicating that greater adherence to the DASH-style dietary pattern was related to better metabolic profiles. However, to fully determine the association of DASH diet with obesity phenotype, further observational and interventional studies are needed to address the role of DASH-style diet in the development of obesity phenotypes and its potential mechanisms
